# Uncovering sex/gender differences of arithmetic in the human brain: Insights from fMRI studies

**DOI:** 10.1002/brb3.2775

**Published:** 2022-09-21

**Authors:** Ting‐Ting Chang, Nai‐Feng Chen, Yang‐Teng Fan

**Affiliations:** ^1^ Department of Psychology National Chengchi University Taipei Taiwan; ^2^ Research Center for Mind, Brain & Learning National Chengchi University Taipei Taiwan; ^3^ Graduate Institute of Medicine Yuan Ze University Taoyuan Taiwan

**Keywords:** arithmetic processing, brain, fMRI, gender differences, mathematical cognition, sex differences

## Abstract

Over the long run, STEM fields had been perceived as dominant by males, despite that numerous studies have shown that female students do not underperform their male classmates in mathematics and science. In this review, we discuss whether and how sex/gender shows specificity in arithmetic processing using a cognitive neuroscience approach not only to capture contemporary differences in brain and behavior but also to provide exclusive brain bases knowledge that is unseen in behavioral outcomes alone. We begin by summarizing studies that had examined sex differences/similarities in behavioral performance of mathematical learning, with a specific focus on large‐scale meta‐analytical data. We then discuss how the magnetic resonance imaging (MRI) approach can contribute to understanding neural mechanisms underlying sex‐specific effects of mathematical learning by reviewing structural and functional data. Finally, we close this review by proposing potential research issues for further exploration of the sex effect using neuroimaging technology. Through the lens of advancement in the neuroimaging technique, we seek to provide insights into uncovering sex‐specific neural mechanisms of learning to inform and achieve genuine gender equality in education.

## Graphical abstract

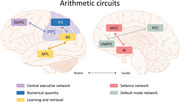

## 1 INTRODUCTION

For the past centuries, scientists and the public have been fascinated by the specificities of sex/gender in mathematical achievement. This question is particularly disputatious, as mathematics plays a crucial role in academic and professional success as well as a career choice and, hence, is highly emphasized in worldwide formal education (Geary, 2013; Ko, 2005; Richland et al., 2007). Although empirical studies have reached the consensus that males and females perform equally well in objective measurements of mathematical performance (Hyde, 2014), there is still a stereotypical view that males outperform females thrive and have a great impact on educational practices, as well as teachers’ and parents’ perceptions. This can lead to severe results in female students avoiding pursuing academic degrees and professional careers. As a result, even to date, there is still significant gender inequality in academic and career participation, especially in the STEM field (Ceci et al., 2014). According to the United Nations Educational, Scientific and Cultural Organization (UNESCO), females only comprised 35% of students enrolled in STEM fields and 28% of researchers in the worldwide populations (UNESCO, 2017). Therefore, scientific evidence on sex/gender differences in mathematical learning must continue to be evaluated to achieve genuine sex/gender equality.

To address the issue, we review behavioral and neuroimaging studies that investigated sex specificity in mathematical learning. Although mathematics is a composite area of knowledge including distinct subdomains, such as arithmetic, algebra, geometry, and calculus, in this review, we focus on arithmetic skills because (i) the vast majority of literature, especially the neuroimaging studies, had focused on arithmetic skills (Chang et al., 2016; De Smedt et al., 2011; Keller & Menon, 2009; Pletzer et al., 2016; Rosenberg‐Lee et al., 2011) as it is the core fundamental component of mathematical knowledge system that involves numbers and its manipulations; (ii) this skill can be settled by primary school stage (Menon et al., 2014); and (iii) individuals with mathematical learning disabilities (MLD) seem to jointly exhibit severe difficulty in arithmetic learning (Butterworth et al., 2011).

With the advancements in non‐invasive methods to image human brain structure and function, the availability of these neuroimaging technologies has provided a novel approach to evaluate the contention of whether sex/gender shows effect by scanning male and female brains. In this review, we will first illustrate behavioral comparisons between males and females focusing on meta‐analytic studies. We will then review how the neuroimaging technique that has long been developed and advanced to understand neuroanatomical and functional mechanisms in cognitive neuroscience—the magnetic resonance imaging (MRI) technique—can be applied to characterize brain response profiles. Next, we will review contemporary studies that have used structural and functional MRI to portray the characteristics of biological sex/gender specificity in the human brain. Finally, we will close this review by proposing perspectives on this critical debate on sex/gender differences that would require further investigation by cognitive and educational neuroscientists. As the definition of sex and gender are topics of great debate such that it is highly difficult to discriminate whether differences between males and females are pre‐wiring by nature or learned from experience, throughout this article, we adopted the term “sex/gender” to capture both the biological bases and the psychosocial expression of masculinity and femininity (Eliot et al., 2021; Jordan‐Young & Rumiati, 2012; Kaiser et al., 2009; Springer et al., 2012). Throughout the review, we demonstrate that males and females are likely to employ divergent neural systems to achieve comparable performance. We seek not only to emphasize specific behavioral and neural mechanisms for each sex, but also to highlight the consequences of the divergent factors that bring to bear the shaping of human learning and cognitive mechanism from biological bases. By utilizing neuroimaging techniques to uncover sex/gender specificity in arithmetic, these findings can potentially be applied globally to reduce sex biases in education.

## 2 DO MALES PERFORM BETTER AT ARITHMETIC?

There is a global stereotype that boys outperform girls in math and science in the long run. This sex‐bias perception existed even when researchers tested college students on implicit measures at an unconscious level (Nosek et al., 2002). Are girls’ math abilities actually below their male counterparts? We seek to answer this question by reviewing behavioral studies that feature comparisons of arithmetic performance between males and females. Taking a massive data approach, we will focus on meta‐analytic studies to culminate these assertions.

The most straightforward method is to compare group differences in averaged arithmetic performance. This approach has yielded various extensive studies that compared math achievements between sexes/genders. An overview of these findings is listed in Table 1. Hyde et al. (1990) adopted meta‐analytic methods in 100 studies encompassing more than three million participants that compared multiple subscales of arithmetic achievements between males and females. The effect sizes were small regardless of the participants' age or the complexity of the arithmetic problems tested (*d* = −0.03 of number concepts, and *d* = 0.08 of arithmetic problem solving). Consistently in a later meta‐analysis of the same author group, arithmetic performance interchangeably favored boys and girls from second grade to high school, but the average effect size remained smaller than 0.01 across all age groups (Hyde et al., 2008). In a more recent study, Lindberg et al. (2010) analyzed 242 contemporary arithmetic performance studies published between 1990 and 2007 that covered 1.2 million participants. Similarly, they found that overall sex/gender differences had also decreased to *d* = 0.05(Lindberg et al., 2010). These results suggested that group comparisons of sex/gender differences in arithmetic performance can seem to be negligible.

**TABLE 1:** Effect sizes of sex difference in mathematics performance

Study	Type	Year	*N*	Age range	Content	*d*
Hyde et al. (1990)	Meta‐analysis	1963−1988	> 3 million	5–55	Math computation	−0.14
					Math concepts	−0.03
					Math problem solving	0.08
Hedges and Nowell (1995)	Large scale	1960–1992	> 0.2 million	15–22	Math	0.16
Hyde et al. (2008)	Meta‐analysis	1970s–1980s	> 7 million	G2–G11	Math skills	< 0.01^†^
Lindberg et al. (2010)	Meta‐analysis	1990–2007	> 1.2 million	Preschool − Adult	Math performances	0.05
	U.S. large data		>1.3 million	7–18	Math performances	0.07
Else‐Quest et al. (2010)	Meta‐analysis	2003	≈ 0.5 million	14–16	TIMSS‐Math	−0.01
					PISA‐Math	0.11
Baye and Monseur (2016)	Large scale	1995–2015	1654	G4, G8, G12	Mean math	−0.06

^†^ The weighted mean of *d* is 0.0065. All *d* s < 0.1 for each grade.

Year = years of the data sets administrated; *N* = number of participants; *d* = mean or weighted effect size. Positive values of *d* represent higher scores for men; negative values of *d* represent higher scores for females; G = grade.

Sex/gender differences in arithmetic performance were also reported to decline with time. In the Hyde et al.’s (1990) study, they separated the studies that were analyzed into two subgroups: those published in 1973 or earlier versus studies published or later. They found that the *d* values for the former were 0.31, while the *d* values declined to 0.14 for the latter, suggesting that the magnitude of male advantage in arithmetic appeared to be reduced across eras. Such a tendency can also be supported by data analyses from global normative surveys that regularly evaluate the classroom performance every 3–4 years, namely TIMSS (Trends in International Mathematics and Science Study). According to the latest TIMSS report, the sex/gender gap favoring eighth grade boys was significantly reduced between 1995 and 2019 in multiple East Asian and western countries, including Australia, England, France, Ireland, Italy, Japan, Korea and Taiwan, and New Zealand (Mullis et al., 2020). These results likely suggested a possible closure of the sex/gender gaps, especially in those gender‐equal countries (Guiso et al., 2008).

Some researchers suspected that sex/gender differences in arithmetic learning are presented on the individual level rather than reflected in the societal group average. Studies suggested that boys have a larger variance in the distribution of math performance than girls, resulting in males being more frequently reported in extreme tails of the distribution (Baye & Monseur, 2016; Maccoby & Jacklin, 1974). In support of this claim, Benbow et al. reported that arithmetic problems involving mathematics reasoning favor males in adolescents, as well as gifted and high‐achieving children (Benbow et al., 2000; Benbow & Stanley, 1980). In the Lindberg et al.’s (2010) study, sex/gender differences were also analyzed based on participants’ ability levels. For the general and low‐ability groups, the effect size of sex/gender difference was only 0.07, but for the highly selective group, the effect size of sex/gender difference was 0.4. On the other hand, by assessing the lower end of the distribution, Barbaresi et al. (2005) reported that male students with low arithmetic achievement tend to deteriorate compared to their female peers. Using large‐scale data from six national data sets, Hedges and Nowell (1995) also found that males showed larger variance in the sampled distribution than females. In order to quantify the existence of sex/gender differences in variability, Lindberg et al. (2010) conducted a method by computing the variance ratio (VR) which divided male variances by female variances. The result was 1.07, leading the authors to conclude that the variance ratio is not far from equal between sexes/genders. The same technique was conducted on larger scale data by Hyde (2014), with the ratios of male to female variance in arithmetic performance compared in multiple meta‐analytic studies. The resulting variance ratio ranged from 1.05 to 1.2. Altogether these results suggested that gender gaps in performance variance are not drastically large and nearly equal (Hyde, 2014; Lindberg et al., 2010).

To summarize, behavioral literature has suggested that the sex/gender gap in arithmetic performance had been negligible and likely diminished over time. It is then intriguing to clarify the neural mechanisms of whether and how each sex/gender learns differently. In the upcoming sections, we will provide a novel technical approach to pursue this issue from the cognitive neuroscience perspective.

## 3 USING NEUROIMAGING TECHNIQUES TO UNDERSTAND SEX/GENDER DIFFERENCES IN ARITHMETIC PROCESSING

In the past decade, an emerging field that aims to provide a linkage between neuroscience and education by applying neuroscience research to educational settings has reached enormous interest. A primary line of research in this field is to probe into the neural mechanism underlying the learning effect. In view of this issue, we will present contemporary studies using neuroimaging techniques to uncover sex/gender‐specific outcomes of brain responses toward arithmetic learning. The cognitive neuroscience approach has provided tremendous insight into understanding biological sex/gender differences. Among the methodologies that measure brain responses, magnetic resonance imaging (MRI) with unprecedented spatial resolution has become one of the primary tools for understanding human brain structure and function. Accordingly, we will focus on structural and functional MRI as advances in this technique have accumulated valuable knowledge to uncover how the brain learns arithmetic (Arsalidou & Taylor, 2011; Chang et al., 2019; De Smedt et al., 2011; Rosenberg‐Lee et al., 2011; Wu et al., 2009). In the following, we first summarize the current understanding of arithmetic‐related neural circuits and then move on to how fMRI can contribute to understanding sex/gender specificity in arithmetic performance.

### 3.1 Arithmetic‐related brain regions

**FIGURE 1:**
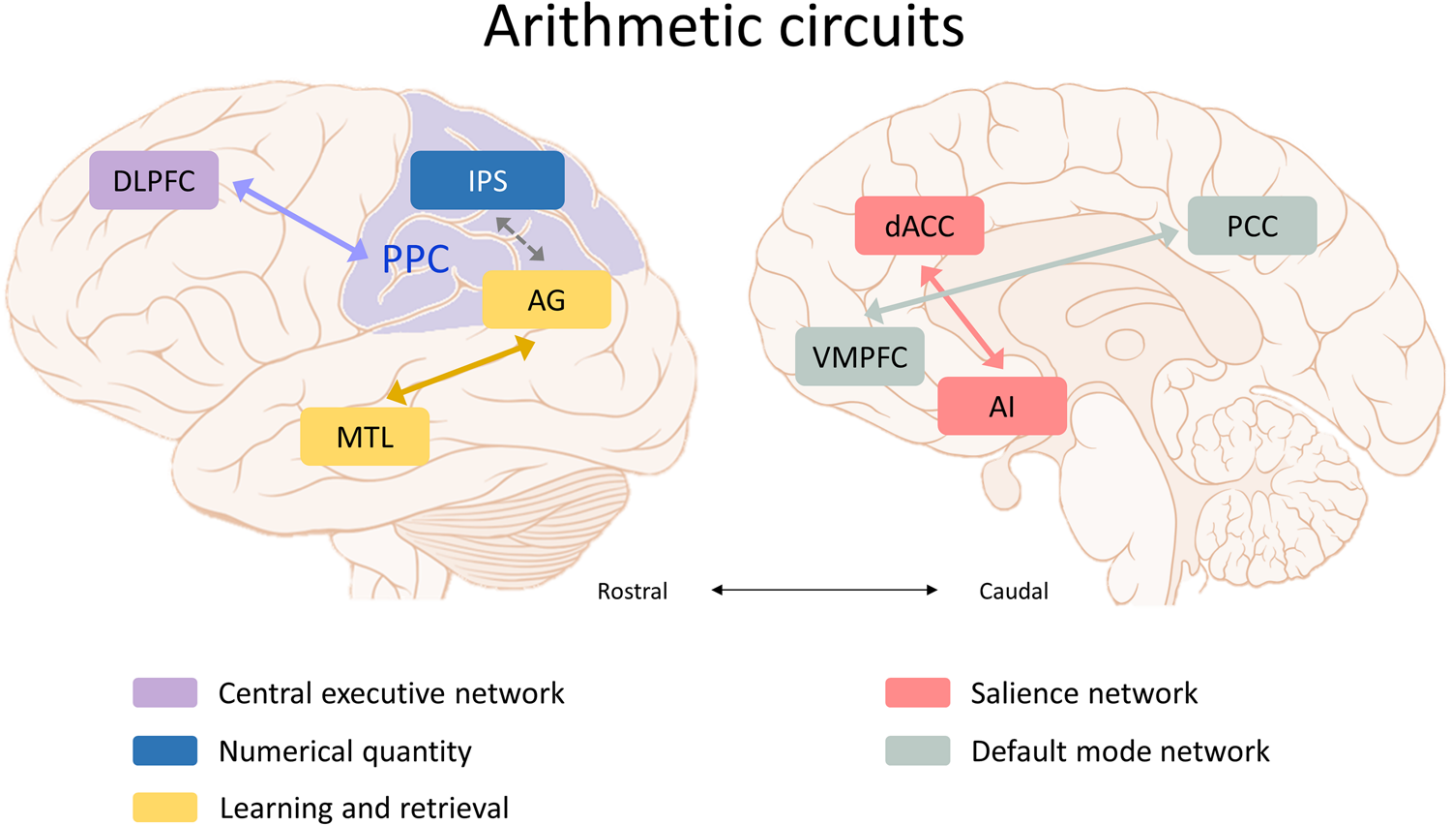
Illustration diagram of the arithmetic circuits. These circuits mainly comprise several nodes within the fronto‐insular‐parietal network, including dorsolateral prefrontal cortex (DLPFC), medial temporal lobe (MTL), dorsal anterior cingulate cortex (dACC), anterior insula (AI), ventromedial prefrontal cortex (VMPFC), and posterior parietal cortex (PPC, shadowed in lavender). The left image shows a lateral view of the brain. Within the PPC subdivisions, intraparietal sulcus (IPS, shown in blue) represents abstract quantity information; and angular gyrus (AG, shown in mustard) is responsible for fact retrieval and generalization during arithmetic problem‐solving. The MTL (shown in mustard), particularly in the hippocampus and parahippocampus, together with the AG, plays an important role in mathematical memory‐based problem‐solving skills. The dorsal frontal‐parietal circuit, PPC, and DLPFC (shown in purple) are critical nodes of the central executive network, maintaining and manipulating information from working memory. The right image depicts a medial view of the brain. The salience network (shown in coral) is predominately anchored in the AI and dACC, and functions by integrating signals and resources to achieve task goals. Posterior cingulate cortex (PCC) and VMPFC are prominent nodes of the default mode network (shown in gray), which are considered to regulate arithmetic processing efficiency.

Before moving into understanding neuroimaging studies of sex/gender differences, we first illustrate the brain regions canonically associated with arithmetic. Since arithmetic skills are the most fundamental mathematics skills that build on the manipulation of core number knowledge, a majority of neuroimaging studies have focused on scanning participants’ brains while performing arithmetic tasks to identify the regions that show the greatest activation levels. This approach has consistently placed a set of distributed brain regions that are activated during arithmetic problem solving (see Figure 1 for illustration). Within this set of networks, the PPC is believed to play the most crucial role in representing and manipulating quantitative information (Ansari, 2008; Cohen Kadosh et al., 2008; Dehaene et al., 2003). Far from being a homogeneous structure, the PPC consists of distinct subdivisions that appear to facilitate specific roles during mental arithmetic (Rosenberg‐Lee et al., 2011; Wu et al., 2009). Within the PPC subdivisions, the IPS, together with its posterior area, are thought to play crucial roles in representing abstract quantity information (Ansari, 2008; Arsalidou & Taylor, 2011; Cohen Kadosh et al., 2008; Dehaene et al., 2003), while the angular gyrus (AG) has been linked to rote fact retrieval while solving more automatic arithmetic problems, such as multiplication (Dehaene et al., 2003; Grabner et al., 2007; Rosenberg‐Lee et al., 2011).

As the human brain is complex and collaged with interconnected nodes, the canonical arithmetic circuits include widespread distributed brain regions outside the PPC, including the anterior insula (AI), the dorsal anterior cingulate cortex (dACC), and the dorsolateral prefrontal cortex (DLPFC) (Cai et al., 2016; Chang et al., 2019; Levy & Wagner, 2011; Ng et al., 2021). The AI coupling with the dACC forms the main components of the salience network (SN) (Menon, 2015b; Seeley et al., 2007). This circuit is involved in the subjective salience of external stimuli and in the contributions to complex cognitive processes, including central executive function and affective processing. The DLPFC, coupled with the PPC, comprises the major nodes of the central executive network (CEN). This set of regions engages in information retention and manipulation during working memory, manipulation of quantities over epochs, building problem solutions, and decision‐making (Chang et al., 2019; Menon, 2015a; Miller & Cohen, 2001; Petrides, 2005; Rottschy et al., 2012). The entire set of the fronto‐insular‐parietal network has been consistently identified when assessing problem‐solving skills, especially those involving numbers and arithmetic, in both children and adults (Arsalidou & Taylor, 2011; Chang et al., 2019; Chang et al., 2016). In a recent fMRI study, Chang et al. (2019) demonstrated that brain response profiles associated with judging sentences that required one‐step arithmetic operations were associated with greater engagement and stronger within‐network connectivity in the fronto‐insular‐parietal circuits relative to judgment over parallel narratives without any numerical information. These circuits were further modulated when the lexical consistency of arithmetic word problem description was tied up with the arithmetic operation of the problem‐solution mathematical model (Ng et al., 2021).

The posterior AG within the PPC coupling with the precuneus typically shows reduced activations during solving the basic arithmetic operation problems (Chang et al., 2016; Grabner et al., 2007; Ischebeck et al., 2006; Rosenberg‐Lee et al., 2011; Wu et al., 2009). Individual differences in performance are associated with AG deactivations, with AG deactivated stronger during more effortful arithmetic tasks and in individuals with poorer arithmetic performance (Grabner et al., 2007; Wu et al., 2009). The PPC and the posterior cingulate cortex (PCC) that showed task deactivations overlapped with prominent nodes of the default mode network (DMN). This network composed of the PPC, PCC, and ventral medial prefrontal cortex (VMPFC) typically activates below baseline when solving difficult tasks (Greicius et al., 2003; Wu et al., 2009). Structural and functional connectivity analyses also demonstrated that the PPC intrinsically correlates with other DMN nodes (Uddin et al., 2010). Using a 2‐year longitudinal design in school‐age children, Wang et al. (2022) found that the arithmetic task‐induced brain activations within the DMN and fronto‐parietal network showed reduced connection to other nodes and became more segregated over time. Collectively, these results suggested the possible role of DMN in regulating arithmetic processing efficiency. The PPC together with the entire set of the DMN was considered to play a domain‐general role during solving effortful math problems rather than serving a math‐specific function (Bloechle et al., 2016; Wu et al., 2009).

The anterior and medial temporal lobules have also been constantly implicated in solving arithmetic problems (Menon et al., 2014). Evidence came from animal models showing that this circuit projected to the prefrontal cortices forms the circuits that are essential for establishing facts in the long‐term memory in the early stage of learning (Squire & Zola‐Morgan, 1991). This model suggested that the parahippocampal‐prefrontal circuit is extra critical for children during the learning stage of arithmetic problems. Consistently, Cho et al. (2011) found that children who solved addition problems using retrieval strategies showed distinct patterns from those who used counting strategies in the parahippocampal‐prefrontal circuits. Brain responses within these circuits are later identified as associated with more efficient retrieval (Cho et al., 2012). The anterior and medial temporal cortices undergo a protracted developmental progression from childhood and transiently upregulate during adolescence to achieve adult‐like performance (Chang et al., 2016; Chang et al., 2015).

Altogether, these results supported that the interconnected nodes jointly engage and synchronize to form the network contributing to the core neural substrates of arithmetic problem‐solving skills, ranging from simple number comparisons to complex arithmetic and problems that require mathematical reasoning (Chang et al., 2019; Cho et al., 2012; Rosenberg‐Lee et al., 2015; Rosenberg‐Lee et al., 2011; Supekar & Menon, 2012). Whether and how the circuits show sex/gender specificity remained to be further explored.

### 3.2 Arithmetic brain networks are modulated by multiple arithmetic constructs

Neuroimaging studies have identified the arithmetic circuits modulated by numerical properties, such as problem difficulty and problem size (Chang et al., 2016; Chang et al., 2015; De Smedt et al., 2011; Metcalfe et al., 2013; Stanescu‐Cosson et al., 2000). The problem size effect indicates that arithmetic problems with larger operand size (e.g., 8 + 7) responded slower and less accurately than problems with smaller size (e.g., 2 + 3). The problem size effect is likely reflecting the specificity of the strategy used in distinct problem types. Small problems are solved by retrieving semantic facts from arithmetic knowledge, whereas large problems are solved by multistep procedural calculation (Barrouillet et al., 2008; Campbell & Xue, 2001; De Smedt et al., 2011). For instance, when performing tasks with different problem sizes, Stanescu‐Cosson et al. found that adults had stronger activations in several regions of the PFC and the bilateral IPS when solving arithmetic problems with large problem sizes than small ones. In contrast, in small problems, inversely, engagements were stronger in the AG than in large problems (Stanescu‐Cosson et al., 2000). Several other studies have also reported similar regions in adults and school‐age children (Chang et al., 2016; Chang et al., 2015; Cho et al., 2012; De Smedt et al., 2011), with the exception that it is the hippocampus rather than the AG that shows stronger activations for small problems (Cho et al., 2012; De Smedt et al., 2011). Together, by providing biological bases, these studies further support the concept of requiring greater involvement of quantity‐based procedural calculation and working memory resources while solving complex problems with larger size and retrieving from rote facts when solving simple problems.

Neuroimaging studies have also shown that arithmetic operations modulate arithmetic circuits. In one adult imaging study, Rosenberg‐Lee et al. demonstrated that solving single‐digit subtraction problems involved greater IPS activation than solving single‐digit addition problems. Chochon et al. (1999) found that multiplication was associated with the left IPS activity, while subtraction was associated with the bilateral IPS activity. A direct comparison of subtraction with multiplication revealed greater activation only in the right IPS. Prado et al. (2011) conducted a quantity comparison task and demonstrated that subtraction was associated with IPS, whereas multiplication was associated with the inferior frontal gyrus (IFG), as well as the middle temporal gyrus (MTG), the language area. These results suggested that subtraction involves a higher level of quantity representation and manipulation, whereas multiplication involves greater verbal fact retrieval. Together, these results point to PPC heterogeneity such that brain responses associated with different arithmetic problem‐solving strategies and numerical properties map onto distinct profiles within PPC regions and their interconnected network. It is still unclear whether and how males and females actively show distinctiveness in the functional architecture during arithmetic processing. In the next section, we attempt to establish the link between sex‐related neuroanatomical patterns and behavioral arithmetic performance.

### 3.3 Sex/gender differences in functional and neuroanatomical brain regions associated with arithmetic brain network

For centuries, whether and how each sex/gender shows distinctiveness in the human brain has been of great interest to neuroscientists, biologists, and physiologists. It was believed that understanding organizational and functional sex/gender differences in human brains could shed light on explaining why males and females exhibited cognitive and behavioral differences (Hyde et al., 1990; Maccoby & Jacklin, 1974; Maeda & Yoon, 2013). Therefore, the dimorphic brains of sex/gender have been extensively examined (cf. Eliot et al., 2021). To align with the objective of this review, we focus on neuroimaging studies that had reported sex/gender specificity in the arithmetic circuits reviewed above; and review empirical studies that compared brain structure and function between males and females using MRI and fMRI methods.

Essentially, current efforts in investigating sex/gender differences in brain structures have moved from small and limited sample sizes to large‐scale mining databases that disseminate in‐depth information about brain structure and function. Many of these investigations have reported region‐specific results favoring either male or female within the arithmetic‐relevant brain network. Table 2 summarizes the examples from the most recent studies measuring regional cortical/subcortical volumes between sexes/genders that include thousands of participants from open access data sets. Many of these studies had identified that MFG and IFG in the prefrontal cortices and the parietal lobe are larger in females. In contrast, the medial temporal subregions, including the parahippocampal gyri, are larger in males, even when total brain volume and body length were taken into account (Fjell et al., 2009; Liu et al., 2020; Lotze et al., 2019; Ruigrok et al., 2014). A more specific example provided by Ritchie et al. (2018) analyzed more than 5000 adult brain structures from UK Biobank (https://www.ukbiobank.ac.uk/). They found that after adjusting total brain volume, there are 13 regions that are larger in females, including MFG and PPC, with the greatest effect identified in the right superior parietal lobe. Males, in contrast, are larger in regions that include MTL as well as the parahippocampal gyri.

**TABLE 2:** Sex difference in neuroanatomical structures within the arithmetic‐related brain circuits

			Regions of differences	
Study	# F/M (*N*)	Age range	Females > Males	Males > Females
Fjell et al. (2009)	676/467	18–94	*n.s*.	Hippocampus
Ruigrok et al. (2014)	1076/1110	7–80	R. MFG, R. IFG, R. Insula, R. OFC, R. IPL, L. pPHG	L. OFC Hippocampus, aPHG
Joel et al. (2015)	495/360	18–79	SFG, Hippocampus,	*n.s*.
Potvin et al. (2017)	1352/1361	18–94	R. MFG, SPL	IFG, OFC, R IPL, R. Insula
Ritchie et al. (2018)	2750/2466	44–77	MFG, SPL, L. IPL,	OFC, R. Insula, PHG
Lotze et al. (2019)	(2838)	21–90	PFC, MFG, OFC, SPL, IPL, Insula.	Hippocampus, PHG
Liu et al. (2020)	488/488	22–35	PFC, MFG, OFC, IPL, SPL, Insula	Hippocampus, PHG

This table includes only studies that reported the frontal‐insular‐parietal and hippocampal regional cortical/subcortical volume differences. All of the results are total brain volume‐ or intracranial volume‐corrected.

Abbreviations: F, females; M, males; n.s., not significant; L, left; R, right; MFG, middle frontal gyrus; IFG, inferior frontal gyrus; OFC, orbitofrontal cortex; IPL, inferior parietal lobule; pPHG, posterior parahippocampal gyrus; aPHG, anterior parahippocampal gyrus; SFG, superior frontal gyrus; SPL, superior parietal lobule; PFC, prefrontal cortex; PHG, parahippocampal gyrus.

Other studies focused on male‐female comparisons over resting state or intrinsic activity/connectivity. Table 3 summarized recent massive analyses over multisite imaging data. Many studies have identified a more significant default mode network connectivity in females. Biswal et al. (2010) conducted three primary analysis methods on resting state scans of 1,093 participants, including seed‐based functional connectivity, fractional amplitude of low‐frequency fluctuation (fALFF), and independent component analysis (ICA). In all the three methods, they reported that females demonstrate stronger PCC connectivity and amplitude. Female advantage in DMN was also identified in several other large‐scale studies using similar techniques (Allen et al., 2011; De Lacy et al., 2019; Ritchie et al., 2018; Tomasi & Volkow, 2012), and the functional connectivity features extracted from DMN are highly predictive of sex/genders (Zhang et al., 2018). Some searchers have associated the female advantage in the DMN with its social function (Ritchie et al., 2018; Zhang et al., 2018) given that females typically perform better on social cognitive tasks such as face recognition (Gur et al., 2012).

**TABLE 3:** Sex difference in resting state fMRI

Study	# F/M (*N*)	Age range (mean)	Measurement	Functional connectivity differences
Biswal et al. (2010)	(1093)	18–68	Seed‐based correlation, ICA, fALFF	Sex differences in various regions and independent networks with divergent directions. F > M generically in DMN.
Allen et al. (2011)	305/298	12–71	Group ICA‐based regression	F > M within DMN; M > F within sensorimotor networks. F > M for intranetwork connections; M > F for internetwork connections.
Tomasi and Volkow (2012)	336/225	18–30	Local functional connectivity density	F > M connectivity densities in DMN, insula, parahippocampal, and inferior parietal.
Zuo et al. (2012)	569/434	(28.1)	Graph theory of network centrality	F > M centrality in hippocampus.
Satterthwaite et al. (2015)	362/312	9–22	Multivariate correlation	M showed greater between‐module connectivity and F showed more within‐module connectivity.
Zhang et al. (2016)	291/203	22–36	Linear regression and graph theory	M > F in the majority of brain regions. M showed higher segregation whereas F showed higher integration.
Ritchie et al. (2018)	2096/1908	(61.6)	ICA‐based estimation	F > M within DMN; M > F between sensorimotor, visual, and rostral lateral prefrontal cortex.
Zhang et al. (2018)	454/366	22–37	Partial least squares regression	DMN exhibited the greatest functional connectivity feature weights to sex/gender discrimination.
De Lacy et al. (2019)	335/335	19–35	ICA‐based estimation	Both F > M or M > F effects were observed in DMN, with an average larger effect size in F.

Abbreviations: DMN, default mode network; fALFF, functional amplitude of low‐frequency fluctuation; ICA, independent component analysis.

Note that finding sex/gender differences in the structure or function of the adult brain by no means implies that the dimorphism of male and female brains is inborn. As the arithmetic circuits undergo protracted development with learning and experience in mathematical cognition from childhood into adulthood (Chang et al., 2016; Chang et al., 2015; Supekar & Menon, 2012), the arithmetic learning systems in human brains show remarkable maturation. Therefore, it is crucial to investigate the accumulated evidence from cross‐sectional and longitudinal studies to reveal how sex/gender differences develop in the brain network and their complex interaction with brain structures across development. Table 4 summarized the current efforts investigating developmental trajectory of sex/gender effect. Again, we emphasize on studies that have reported age by sex interaction. Most of these current efforts have steadily searched for structural differences in 8‐ to 30‐year‐olds. Satterthwaite et al. (2014) assessed 922 children and young adults using arterial spin‐labeled MRI measuring blood perfusions. They found that males showed a linear decline with age in the PPC, PFC, and lateral temporal cortices. In contrast, females showed an inverted u‐shaped pattern between this age range (i.e., initially declined in childhood until an increase in adolescence). Using the same data set, Gennatas et al. (2017) reported that males have a larger cortical thickness in the bilateral insula until age 12 and in frontal and occipital lobes until age 15. Afterward, the sex/gender difference reverses, leading to a female advantage after adolescence in these subdivisions. Koolschijn and Crone (2013) investigated brain volumes and cortical surface of 442 typically developing individuals with a similar age range. They found that males showed larger surface area in fronto‐parietal and temporal lobe between age of 8 to 15, whereas females are relatively stable with the age increase. Although most of these developmental studies are limited by the sample distributions skewed toward age 18 and beyond, it is clear that brain maturation shows a different progression between sexes/genders. Substantial examination of samples evenly distributed across all ages with both cross‐sectional and longitudinal design remained needed to fill the major gap in the developmental progression of lifespan, including both the immature and the aging end.

**TABLE 4:** Sex differences in brain development

Study	# F/M	Age range	Measurement	Developmental trajectory differences
Mutlu et al. (2013)	69/68	6–30	Cortical thickness	F > M thinning rate in the superior frontal, orbitofrontal, SMG, and temporal regions
Koolschijn and Crone (2013)	223/219	8–30	Gray matter volume	M > F general volume decrease
			Cortical thickness	*n.s*.
			Cortical surface	M > F greater surface contractions in frontal, parietal, and temporal cortex
Satterthwaite et al. (2014)	518/404	8–22	Cerebral blood flow	In DLPFC, VMPFC, Insula, IPL, and hippocampus, declined in M until late adolescence, whereas F declined until mid‐adolescence but increased thereafter.
Gennatas et al. (2017)	648/541	8–23	Gray matter density	*n.s*.
			Cortical thickness	M > F in insula thickness until age 12, and in frontal and occipital until age 15; thereafter, the effect reverses, resulting in F > M
Wierenga et al. (2019)	144/127	8–26	VR in thickness	M > F in mOFG, precentral gyrus, temporal pole, and occipital F > M in insula and PCC
			VR in surface	M > F in insula, PCC, and precentral gyrus F > M in ACC and SMG
Forde et al. (2020)	1707/1362	8–95	VR in thickness	*n.s*.
			VR in surface	M > F in most age populations, whereas F > M in oldest populations (aged > 75–80)

Abbreviations: ACC, anterior cingulate cortex; DLPFC, dorsolateral prefrontal cortex; IPL, inferior parietal lobule; mOFG, medial orbitofrontal gyrus; n.s., not significant; PCC, posterior cingulate cortex; SMG, supramarginal gyrus; VMPFC, ventromedial prefrontal cortex; VR, variance ratio, the male variance divided by the female variance.

In sum, although the effect sizes of these above‐reviewed studies were considered small and varied with neuroimaging data processing tools, these studies do agree on the possible separation between males and females in the wired arithmetic learning‐associated brain circuits and further suggest that sex/gender differences are likely in nature and show complementarity. More imaging studies of how these sex/gender‐related brain patterns correlate with cognitive functions may help explain the debate about sex/gender differences in arithmetic learning. In light of this, we will review studies that directly measure brain response profiles during active engagement in arithmetic tasks in the next section.

### 3.4 Sex/gender differences in brain regions during arithmetic task fMRI

To date, most task fMRI studies assessing sex/gender differences in brain responses have focused on linguistic stimuli, visuospatial tasks, and emotion processing (cf. (Eliot et al., 2021), but extremely few studies had systematically investigated sex‐/gender‐specific brain effects in relation to arithmetic problem processing. As such, here we highlight the current studies using arithmetic task fMRI to examine cognitive and biological differences between males and females.

Table 5 summarizes the studies that evaluate the brain response profiles of males and females while performing arithmetic tasks within MRI scanners. To date, only four studies have directly investigated sex/gender effects associated with arithmetic tasks. The first attempt was conducted by Wang et al. (2007) who compared sex/gender differences in brain responses underlying high‐pressured serial subtraction of 13 from a 4‐digit number versus counting backward from 1000 without pressure. They found that males showed stronger activations in the right PFC during the stressed task. Keller and Menon (2009) were the first to use both structural and functional MRI to compare neuroanatomical and neurofunctional sex/gender differences. When healthy adult participants evaluated the correctness of 3‐operand single‐digit equations mixed with addition and subtraction, males were reported as engaging a greater level of the posterior visual stream, including the right IPS, AG, ventral temporal occipital cortex, and parahippocampal gyri compared to females. Paradoxically, structural data computed on regional density and volume of the brain revealed a reverse pattern; that is, females showed higher density and volume in these regions than males. In another study, Pletzer et al. (2016) examined brain activations of young adults while performing 2‐operand subtraction and multiplication tasks in MRI scanners. They identified the conventional operation effect by which subtraction elicited stronger IPS activations than multiplication, and multiplication engaged in less AG deactivations than subtraction as in previous literature (Chochon et al., 1999; Prado et al., 2011; Rosenberg‐Lee et al., 2011). Critically, Pletzer et al. (2016) found that this operation effect was more salient in males, with males displayed stronger IPS activations and fewer AG deactivations.

**TABLE 5:** Sex differences in brain responses during performing numerical tasks

					Regions of differences	
Study	# F/M	Age (SD)F/M	Task	Perform. Diff.	Females > Males	Males > Females
Wang et al. (2007)^†^	16/16	22.8 (2.4)/ 24.3 (3.1)	Serial subtraction	*n.s*.	PCC	R. PFC, R. AG
Keller and Menon (2009)	25/24	24.4(4.5)/23.5(4.9)	3‐operand equation	*n.s*.	*n.s*.	R. IPS, R. AG, R. LG, R. PHG
Pletzer (2016)	34/40	25.6(4.3)/25.3(4.7)	2‐operand equation	*n.s*.	*n.s*.	*Subtraction*: L. IPS, L. SMA, ACC, Insula, L. postcentral gyrus, R. precentral gyrus *Multiplication*: L. postcentral gyrus *Subtraction > multiplication* mPFC/ACC, SMA, L. IPS, R. Insula, R. precentral gyrus
Kersey et al. (2019)*	Children 55/49	(range) 3–10	Natural viewing	*n.s*.	*n.s*.	*n.s*.

^†^ Study that measures cerebral blood flow.

* Study that measures neural similarity and neural maturity.

Abbreviations: L, left; R, right; n.s., not significant; Perform. Diff., performance differences; ACC, anterior cingulate cortex; AG, angular gyrus; IPS, intraparietal sulcus; LG, lingual gyrus; mPFC, medial prefrontal cortex; PCC, posterior cingulate cortex; PFC, prefrontal cortex; PHG, parahippocampal gyrus; SMA, supplementary motor area.

Although being less consistent due to the varying task and difficulty, all these current efforts have indicated that the fronto‐parietal arithmetic‐related circuits tend to show sex/gender specificity. Note that all these studies have shown that males and females were equivalent in performance levels, given the distinct brain response profiles, particularly in the PFC and PPC. These results suggested that males and females engaged in two complementary but equally successful systems, at least while performing arithmetic tasks. The sex/gender‐specific fronto‐parietal activations were attributed to problem‐solving strategy difference (Keller & Menon, 2009; Pletzer et al., 2016), for example, in using visuospatial strategies. Both Thomsen and Weiss found that males showed stronger activations in the IPS, whereas females showed increased activations in the right inferior frontal gyrus (Thomsen et al., 2000; Weiss et al., 2003) during performing mental rotation tasks without performance differences (Thomsen et al., 2000). On the other hand, women tend to avoid using spatial strategies (Postma et al., 2004). Sex/gender effect in mental rotation tasks favoring males can be large as *d* = 1.03, especially under high‐pressure conditions (Voyer, 2011). It is likely that males and females show specificity in neural resources used to solve strategy‐specific arithmetic problems to achieve equivalent performance.

Advances in fMRI techniques also shed lights on modeling sex/gender profiles. A more recent study conducted by Kersey et al. (2019) computed neural representational similarity. Unlike conventional methods, which emphasize localization of brain responses, this method probes spatial correlation in activity patterns associated with distinct stimuli. Kersey et al. investigated developmental progression by measuring brain response profiles of 3‐ to‐10‐year‐old children using a naturalistic approach while children watched mathematics‐scene education videos. The intersubject correlations over brain responses were computed across all children to obtain the index of neural similarity (child‐to‐child correlation) and neural maturity (child‐to‐adult correlation) across individuals. The results showed that both girls and boys demonstrated high neural similarity as well as neural maturity. This study provides interpretation of sex/gender effect from the perspective of similarity, rather than discriminability, with state‐of‐the‐art multivariate fMRI technology. The approach provides useful knowledge to uncover brain organizations and warrants primary research efforts.

## 4 SUGGESTED FUTURE DIRECTIONS AND CONCLUSIONS

The brain‐based biological mechanisms of sex/gender specificity shall continue to be explored. It is crucial to provide unique perspectives using state‐of‐the‐art neuroimaging techniques to understand the specificity of biological sex/gender in the human brain. As such, in the final section, we propose several potential research directions for future studies to uncover the brain profile of each sex/gender. The first potential research area is to further investigate the biological mechanisms of sex/gender differences, in particular, how hormone levels affect each sex/gender in arithmetic processing. Sex differentiation generally begins at conception during fertilization within the maternal environment since sex chromosomes determine the biological sex of each individual. The level of sex hormone to which the embryo is exposed then controls sexual development (Wilson et al., 1981), contributes to the modulation of neural mechanisms on cognitive and behavioral development (Collaer et al., 2002; Collaer & Hines, 1995), and upregulates internal function across puberty. Pletzer et al. (2011; 2013) made the first attempts to measure brain responses toward a number bisection task and a multidigit comparison task during women in different menstrual cycle stages. They found that women made more errors and more enhanced brain responses in the PFC and DMN during the early follicular phase when estrogen and progesterone levels are low. These results have suggested that brain and neural mechanisms can be tightly linked with hormone levels determined at birth. However, both studies were constrained by the small sample size (15/16 in each sex group), greatly limiting the interpretability of the findings. How hormone levels affect the arithmetic performance of each sex and furthermore, at different developmental stages throughout the lifespan, especially during adolescence, remained critical for further investigations.

An extended approach we advocate is to map male and female brains onto a continuum rather than frame the brain as sexual dimorphism, as genetic‐ and hormone‐level effects can also be beyond binary. Although most current studies on sex differentiation had characterized sex labels based on the binary classification of sex chromosomes, sex is much more complicated than it was considered. A genetically defined male can have a female gonad and genital (Ainsworth, 2015). An emerging view thus considers sex as a spectrum rather than a dimorphism. Investigating sex‐specific brain mechanisms with a dimensional approach of masculinity/femininity rather than the dichotomy of male/female can be more promising for future research.

Identifying the context‐dependent sex/gender specificity shall continue to be an important approach, as individual differences in sex/gender differences have been consistently reported. For example, the Lindberg et al.’s (2010) study suggested females performed better on algebra problems (*d* = –0.32) whereas males were more accurate on items that assess measurements (*d* = 0.40). Literature also reported that spatial ability, such as mental rotation, sex/gender effect favoring males can be large as *d* = 1.03, especially during short time‐limited conditions (Voyer, 2011). Identifying the mechanisms of when and how each sex/gender shows specialties, from both cognitive and neural perspectives, must continue to be examined.

Cultural input is another robust factor that can lead to the sex/gender bias. Multiple cross‐national studies such as PISA have shown that the more gender‐equal the culture is, the fewer females would underperform in arithmetic than males (Brown & Alexandersen, 2020; Else‐Quest et al., 2010; Eriksson et al., 2020; Guiso et al., 2008), suggesting that sex/gender differences in arithmetic performance can be enlarged in those less gender‐equal countries (Guiso et al., 2008). Together, these results indicated that sex/gender‐specific effect can exist under certain conditions. This inequality is highly susceptible to societal perceptions. Cultural factors can enhance the gender gap, such that sex/gender differences in global arithmetic performance might not be about sex/gender in nature but the expected role in society. The neural mechanisms on how culture and learning environment affect sex/gender remain crucial in pursuing the field.

The newly developed fMRI statistical techniques can also provide innovations to the endeavor in the field. With the emerging literature on neuroimaging methods in the past decades, neurofunctional mapping of the human brain has switched from univariate analysis aimed at localizing certain regions associated with specific cognitive function to multivariate methods seeking brain response patterns and neural circuits comprised of multiple distributed parts (Bressler & Menon, 2010; Menon, 2015a; Uddin et al., 2010). Coupled with contemporary large‐scale open‐access neuroimaging databases, the comparison of male and female brains using brain connectivity, machine learning algorithm, and other multivariate methods have been extensively implemented and published in recent years (Eliot et al., 2021). The aggregation of massive data coupling with state‐of‐the art analysis methods has created a new benchmark for measuring neurofunctional brain morphometry. To achieve the goal, Bethlehem et al. (2022) accumulated more than 100 primary MRI studies that included more than 100,000 human participants aged from 0 to 100 years and, for the first time, constructed the centile score of brain charts for human lifespan by fitting the data with sex stratified and age as a function. Although ethnicity and age group diversity remained issues to be solved in this study, it provides a vigorous benchmark of normative developmental progression in understanding the hallmark of the human brain.

To conclude, although women remain minorities and underrepresented in STEM fields, comparisons between male and female performance suggest that sex/gender differences in arithmetic behavioral performance gradually diminished over time. This intellectual equality was achieved by highly complicated brain‐based biological mechanisms that vary across measurements, developmental progression, and even analyzing protocols. These results suggested that each sex/gender used a distinct profile to achieve parallel performance. Thus, behavioral assessments may not always secure sex/gender similarity at the cognitive processes level. Neuroimaging utilities, in contrast, have a strong potential to provide useful knowledge that is unseen in behavioral results alone.

Although the sex/gender‐biased male‐math stereotype is still prevalent, a growing endeavor has been dedicated to encouraging females to pursue STEM careers. Based on neuroimaging techniques developed in past decades, which provided remarkable insights into uncovering the human neural mechanism, here, we have reviewed how sex/gender differences associated with arithmetic in the human brain can be measured. Despite the fact that more efforts are needed to clarify the existing literature, we seek to promote using novel imaging techniques to uncover new evidence of sex/gender difference/similarity profiles that are necessary to fully characterize mathematical cognition, human learning mechanisms, and education to achieve genuine equality between men and women.

## CONFLICT OF INTEREST

The authors declare no conflicts of interest.

### PEER REVIEW

The peer review history for this article is available https://publons.com/publon/10.1002/brb3.2775.

## Data Availability

Data sharing not applicable to this article as no data sets were generated or analyzed during the current study.
